# Homeopathy is not placebo effect: proof of the scientific evidence for homeopathy in open access trilingual e-book

**DOI:** 10.1016/j.clinsp.2024.100456

**Published:** 2024-08-03

**Authors:** Marcus Zulian Teixeira

**Affiliations:** Departamento de Psiquiatria, Faculdade de Medicina, Universidade de São Paulo, São Paulo, SP, Brazil

## Abstract

• Homeopathy is based on four heterodox and complementary scientific assumptions.
• Pseudoskeptics deny these assumptions and any scientific evidence that proves them.
• The e-book proves the scientific evidence for homeopathy.
• The e-book describes hundreds of experimental and clinical studies in homeopathy.
• The e-book demystifies the fallacy that “homeopathy is placebo effect”.

Homeopathy was founded in 1796 by the German physician Samuel Hahnemann. It is an integrative medical practice that uses a clinical approach based on four heterodox and complementary scientific assumptions (principle of therapeutic similarity, homeopathic pathogenetic experimentation, use of individualized medicines and in dynamized doses), with the aim to awaken a healing response from the body against its own disorders.^1^

In addition to these scientific premises, the homeopathic epistemological model also uses vitalist and miasmatic philosophical conceptions to expand understanding of the complex process of human illness, attributing to the imbalance of the organic vital force and the manifestation of chronic miasms the primary and fundamental causes of the diseases, respectively. Supported by conceptual, functional and experimental correlations, this homeopathic vital force would find its representation or biological substrate in the genome (exome plus epigenome), while the chronic miasms would be biologically represented by the disease-promoting epigenetic alterations.^2^, ^3^, ^4^, ^5^

Because it is based on epistemological premises that are different from conventional medical practice, homeopathy is generally poorly understood, suffering criticism from prejudiced individuals who systematically deny homeopathic principles and any scientific evidence that proves them. In reality, they are pseudoskeptics masquerading as pseudoscientists.^6^

In order to enlighten everyone and demystify culturally ingrained pseudoskeptical fallacies (such as, “there is no scientific evidence for homeopathy” and “homeopathy is placebo effect”), the Technical Chamber for Homeopathy of the Regional Medical Council of the State of São Paulo (Cremesp) produced the Special Dossier “Scientific Evidence for Homeopathy” in 2017, available in open access trilingual editions (Portuguese, English and Spanish) in the *Revista de Homeopatia* (São Paulo) and *La Homeopatía de México* scientific journals.^7^, ^8^, ^9^

This Dossier is composed of nine narrative research reviews in several medical science fields (historical, social, medical education, pharmacological, basic, clinical, patient safety and pathogenetic experimentation), encompassing hundreds of scientific articles describing experimental and clinical studies. It seeks to highlight the state of the art of homeopathic research.^7^, ^8^, ^9^

Then, in order to expand and update this scientific evidence for homeopathy, the authors published the electronic book (e-book, PDF editing) in Portuguese “*Homeopatia não é efeito placebo: comprovação das evidências científicas da homeopatia*” in 2023,^10^ which was translated into English and Spanish in 2024 *(“Homeopathy is not placebo effect”: proof of scientific evidence for homeopathy / “La homeopatía no es efecto placebo”: comprobación de las evidencias científicas en homeopatía)*, being made available in open access editions in the Virtual Health Library (VHL-LILACS-BIREME)^11^, ^12^, ^13^ and in the USP Open Books Portal,^14^^,^^15^ increasing knowledge of the area in 13 interactive chapters.

In turn, this trilingual book series were also made available in EPUB editing (Kindle, Amazon)^16^, ^17^, ^18^ to expand the dissemination scope of the material, and we are inviting homeopaths from other countries to collaborate in translating it into other languages.

Starting the work, the chapter “Homeopathy” discusses the epistemological premises of the homeopathic model (principle of therapeutic similitude, homeopathic pathogenetic experimentation, use of individualized medicines and in dynamized doses)^1^ in detail, describing its evidence in general and providing the reader with an overview of treatment and clinical practice in homeopathy.

Then in the chapter “Clinical epidemiology in homeopathy”, the principles of homeopathic clinical epidemiology are addressed after a review of the principles of classical clinical epidemiology and the types of epidemiological studies used to evaluate the efficacy and effectiveness of conventional treatments, as well as the types of epidemiological studies in homeopathy. It is worth highlighting that the epistemological premise of “individualization of homeopathic treatment in view of the symptomatic totality characteristic of the patient-disease binomial” is a *sine qua non* condition for dynamized homeopathic medicine (ultra-diluted and with infinitesimal pathogenetic power) to be able to awaken a curative response.^19^

Next, in addition to general databases (LILACS and PubMed), several specific homeopathic databases are described in the chapter “Overview of homeopathy research – Databases”; these databases group together a wide range of homeopathic studies indexed in the areas of basic and clinical research, from experimental studies in biological and physicochemical models (Homeopathy Basic Research Experiments database, HomVetCR database and PROVINGS.INFO database) through to epidemiological clinical studies of all types (Clinical Outcome Research in Homeopathy, Homeopathic Intervention Studies and CAM-QUEST databases).

The principle of similitude (similarity) is approached according to the homeopathic model and modern pharmacology in the chapter “Pharmacological basis of the principle of similitude”, describing hundreds of experimental and clinical studies that support the curative response of homeopathic treatment (vital reaction or therapeutic similarity) in accordance with manifestations of the rebound effect of modern drugs (paradoxical reaction of the organism).^20^, ^21^, ^22^ Furthermore, it describes the proposal to use modern drugs according to the principle of therapeutic similitude, using the rebound effect of drugs curatively.^23^, ^24^, ^25^

In the field of basic research in homeopathy, the chapter “Experimental studies in biological models (*in vitro*, plants and animals)” describes hundreds of experimental studies in cells, plants and animals that demonstrate the superiority of homeopathic medicine over control groups, highlighting that “homeopathy is not placebo effect” in systematic reviews and meta-analyses.^26^, ^27^, ^28^

In the field of clinical research in homeopathy, the chapter “Randomized Controlled Clinical Trials (RCTs)” describes dozens of randomized, double-blind and placebo-controlled clinical trials (level of evidence 1B) of good methodological quality which demonstrate the effectiveness of the homeopathic treatment versus placebo. Four chapters address systematic reviews of RCTs, global (any clinical indication) and specific (specific clinical indication) with and without meta-analyses, increasing the level of evidence (1A) of the clinical effectiveness of homeopathy.

In the chapter “Systematic reviews and global reports with positive results of homeopathy compared to placebo”, five global systematic reviews of RCTs with meta-analyses are described which demonstrate the superiority of homeopathic treatment over placebo.^29^, ^30^, ^31^, ^32^, ^33^ On the other hand, two global systematic reviews of RCTs, one with meta-analysis and the other without, are described in the chapter “Systematic reviews and global reports with negative results of homeopathy compared to placebo (Methodological flaws)”. These presented negative results of homeopathy compared to placebo,^34^^,^^35^ however numerous biases and methodological flaws in the studies are evidenced, as demonstrated in several post-hoc analyses published later.

Confirming these post-hoc analyses, a systematic review of global systematic reviews of RCTs with meta-analyses described previously was published in 2023, demonstrating that “global systematic reviews of homeopathic RCTs with meta-analyses reveal significant positive effects of homeopathy compared to placebo”, and that “there was no support for the alternative hypothesis of no outcome difference between homeopathy and placebo”.^36^

Specific systematic reviews are described in the chapter “Systematic reviews for specific clinical conditions”, which demonstrated the superiority of homeopathy over placebo in several clinical conditions: with meta-analyses (allergic rhinitis, acute childhood diarrhea, postoperative ileus and attention deficit disorder with hyperactivity) and without meta-analyses (acute otitis media, postoperative inflammation, psychiatric disorders and rheumatic diseases).

Then in the chapter “Observational studies”, analytical observational studies (level of evidence 2B) were mainly addressed, describing robust cohort studies that presented important information about the effectiveness and cost-effectiveness of homeopathic treatment in thousands of patients in the long term and in various clinical conditions.^37^, ^38^, ^39^, ^40^

Concluding the book, the chapter “Pseudoskeptical and pseudoscientific strategies used in attacks on homeopathy” discusses pseudoskepticism and pseudoscience, describing the tell-tale signs of pseudoskepticism (bogus skepticism or pathological skepticism) in detail, which is a topic of fundamental importance for unmasking pseudoskeptics and pseudoscientists who systematically deny the vast amount of scientific evidence for homeopathy cited throughout the work.^6^

Thus, despite the difficulties and limitations that exist in developing research in homeopathy, whether due to methodological aspects or the lack of institutional and financial support, the significant set of experimental and clinical studies described in the book is indisputable proof that “there is scientific evidence for homeopathy” and that “homeopathy is not placebo effect”, contrary to the falsely disseminated prejudice. However, new studies must continue to be developed to improve clinical practice and elucidate peculiar aspects of the homeopathic paradigm.

## Funding

This research did not receive any specific grant from funding agencies in the public, commercial, or not-for-profit sectors.

## Conflicts of interest

The authors declare no conflicts of interest.
